# Emerging Role of Dipeptidyl Peptidase-4 in Autoimmune Disease

**DOI:** 10.3389/fimmu.2022.830863

**Published:** 2022-03-04

**Authors:** Jie Huang, Xinxin Liu, Yingying Wei, Xinlu Li, Shupei Gao, Lingli Dong, Xiaoquan Rao, Jixin Zhong

**Affiliations:** ^1^Department of Rheumatology and Immunology, Tongji Hospital, Tongji Medical College of Huazhong University of Science and Technology, Wuhan, China; ^2^Department of Cardiovascular Medicine, Tongji Hospital, Tongji Medical College of Huazhong University of Science and Technology, Wuhan, China

**Keywords:** autoimmune, autoinflammatory, DPP4, inflammation, dipeptidyl peptidase

## Abstract

Dipeptidyl-peptidase IV (DPP4), originally identified as an aminopeptidase in 1960s, is an ubiquitously expressed protease presented as either a membrane-bound or soluble form. DPP4 cleaves dipeptide off from the N-terminal of its substrates, altering the bioactivity of its substrates. Subsequent studies reveal that DPP4 is also involved in various cellular processes by directly binding to a number of ligands, including adenosine deaminase, CD45, fibronectin, plasminogen, and caveolin-1. In recent years, many novel functions of DPP4, such as promoting fibrosis and mediating virus entry, have been discovered. Due to its implication in fibrotic response and immunoregulation, increasing studies are focusing on the potential role of DPP4 in inflammatory disorders. As a moonlighting protein, DPP4 possesses multiple functions in different types of cells, including both enzymatic and non-enzymatic functions. However, most of the review articles on the role of DPP4 in autoimmune disease were focused on the association between DPP4 enzymatic inhibitors and the risk of autoimmune disease. An updated comprehensive summary of DPP4’s immunoregulatory actions including both enzymatic dependent and independent functions is needed. In this article, we will review the recent advances of DPP4 in immune regulation and autoimmune rheumatic disease.

## Introduction

Dipeptidyl-peptidase IV (DPP4), also known as CD26, was first discovered as a protease in 1966 (1). DPP4 is mainly expressed on the cell surface, forming a homodimer. It is widely expressed on epithelial cells in various tissues (kidney, bile ducts, liver, lung and intestine), some endothelia cells, leukocyte subsets and fibroblasts (2, 3). The full length of human DPP4 is 766 amino acids (AA), including a short 6-AA cytoplasmic tail, a 22-AA transmembrane hydrophobic segment, and a 738-AA extracellular portion (2, 4, 5). In addition to the membrane-bound form, DPP4 can also be cleaved off from the cell membrane and released into plasma and other body fluids, forming a soluble form that lacks cytoplasmic domain and transmembrane domain. Since the catalytic domain is located in the extracellular portion, soluble DPP4 maintains the enzymatic activity (2).

The substrates of DPP4 have a unique feature of amino acid sequence: with alanine or proline as the preferred residue at the second amino acid. The substrates of DPP4 are categorized into incretin peptides, chemokines and cytokines, and neuropeptides. By cleaving X-Pro or X-Ala dipeptides off from the N-terminal, DPP4 regulates the biological function of its substrates. For example, DPP4 converts glucagon-like peptide-1 (GLP-1) (7–36) and (7-37) into inactive forms GLP-1(9-36) and GLP-1(9-37), which are unable to bind GLP-1 receptor and induce insulin release from pancreatic β cells (6).

Later studies identified DPP4 as the adenosine deaminase (ADA) binding protein. By transducing costimulatory signal in T cells upon stimulation with ADA, DPP4 is considered as a T cell activation marker (7). In 2013, DPP4 was discovered as an entry receptor for Middle East Respiratory Syndrome Coronavirus (MERS-CoV) (8). By binding to spike protein on the surface of MERS-CoV, DPP4 expressed on the epithelial cells in respiratory system mediates the entry of the virus into the host cell (9–11). In addition, DPP4 had also been identified as a co-receptor for human immunodeficiency virus (12). However, later studies indicate that CCR5 is the major co-receptor for the entry of human immunodeficiency virus into CD4^+^ T cells (13, 14) The selective expression of CCR5 on DPP4^+^ T cell subsets may partially explain the association between HIV infection and DPP4 expression (15). A recent study identified a novel implication of DPP4 in scarring and wound healing (16). The author discovered a subpopulation of fibroblast expressing DPP4 is responsible for the bulk of connective tissue deposition in dermal scars. Inhibition of DPP4 reduced scar formation in a mouse model of wound healing. Another study reported that a mesenchymal progenitor cell population expressing DPP4 displayed a highly proliferative and multipotent phenotype and regulated the differentiation of adipocytes (17).

Autoimmune diseases are a group of chronic disorders characterized by autoimmune-mediated damage in multiple systems. Elevation of diverse inflammatory cytokines or chemokines, along with activation of multiple immune cells, could be observed in patients with autoimmune disease. In a number of autoimmune diseases, such as systemic sclerosis and IgG4-related disease, fibrosis also plays a critical role in their pathogenesis. With its involvement in immune regulation and fibrosis, DPP4 may have a pivotal implication in the development of autoimmune disease. Clinical evidence has suggested an association between the use of DPP4 enzymatic inhibitors and several autoimmune disorders, which is summarized by Zhao group and Sahoo group in 2014 and 2021 respectively (18, 19). While DPP4 has been considered a moonlighting protein due to its multifunctional features in different types of cells, an updated comprehensive summary of DPP4’s immunoregulatory actions including both enzymatic dependent and independent functions is needed. This review focuses on emerging evidence of DPP4 in immune regulation and attempts to build a bridge between DPP4 and autoimmune diseases.

## The Role of DPP4 in Immune System

**Figure 1:**
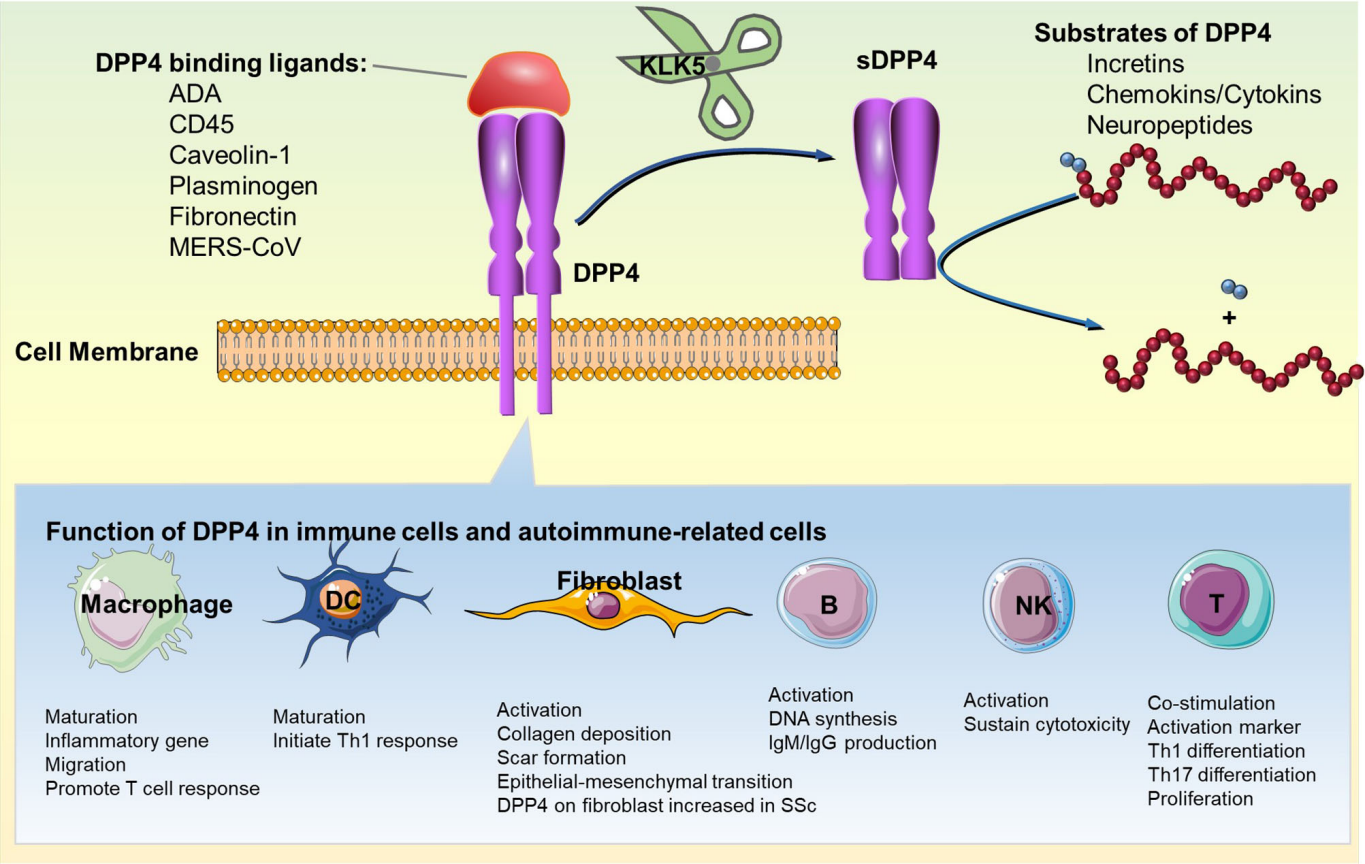
Immunoregulatory function of DPP4: enzymatic and non-enzymatic functions of DPP4 in immune cells and fibroblast are shown. DC, dendritic cell; DPP4, dipeptidyl peptidase-4; IgG, immunoglobulin G; IgM, immunoglobulin M; KLK5, kallikrein-related peptidase 5; sDPP4, soluble DPP4; Th1, type 1 helper T cell; Th17, type 17 helper T cell.

DPP4 is expressed in many types of immune cells, including T cells, B cells, natural killer cells (NKs), dendritic cells (DCs), and macrophages (**Table 1**) (36). The expression level of DPP4 is also associated with the activation status of immune cells. Both enzymatic dependent and independent functions of DPP4 are involved in the regulation of immune function (**Figure 1**).

**Table 1:** The expression and function of DPP4 in immune cells.

	Expression	DPP4 Function	References
CD4^+^ T cells			
Th1	High expression	Co-stimulation	(20)
Th2	Relative low expression	Elevated DPP4 expression was associated with the production of Th2 cytokines	(20, 21)
Th17	High expression	Co-stimulation, correlated with Th17 cytokine production	(22, 23)
Treg	Low expression		(24)
CD8^+^ T cells	High/negative expression	Co-stimulation	(25, 26)
B cells	Low expression	Co-stimulation, promote DNA synthesis, Ig production, and Ig isotype switching	(27, 28)
DCs	Positive expression	Modulate adenosine concentration by DPP4/ADA interaction, recruit Th1	(29, 30)
NK	Low expression	Co-stimulation, maintain cytotoxicity	(31, 32)
Macrophage	Positive expression	Regulatie M1/M2 macrophage polarization	(33, 34)
Fibroblast	Specific subpopulation	Activation marker	(16, 35)

### T Cells

The expression of DPP4 in T cells is variable in different subpopulation and tightly regulated by the level of cell activation (37). Previous research suggested that both Th1 and Th2 express DPP4 on the surface, whereas Th1 cells express significant higher amount of DPP4 than Th2 cells (20). Flow cytometry analysis of DPP4 expression in different CD4^+^ T helper subsets shows that the expression of DPP4 is highest in Th17 compared to Th1 and Th2 (22). On the contrary, DPP4 expression is low on Treg cells and has been regarded as a negative regulator of Treg function (38). In CD8^+^ T cells, by providing co-stimulatory signal, DPP4 represents a specific marker of successful memory development (25, 26).

During the thymus maturation, DPP4 is considered as a maker of differential regulation of human lymphocytes activation (39). DPP4-deficient animal models show decreased number of overall lymphocytes and decreased proportion of CD4+T cells and memory T cells, while naïve T cells relatively increase (27, 40, 41).

In *in vitro* studies, inhibition of DPP4 induced immunosuppressive cytokine TGF-β, but decreased production of proinflammatory cytokines including IL-2, IL-6, and IFN-γ (41–43). There was a debate that DPP4 only exerts T cells activation function *via* its enzymatic activity (44, 45). However, later studies confirmed that DPP4 may also promotes T cell activation and proliferation by enzymatic independent actions. DCs, as most potent antigen-presenting cells(APCs), are pivotal in directing activation and differentiation of naïve T cells (46, 47). DPP4 and some adenosine receptors, including A_1_R, A_2A_R and A_2B_R, serve as binding proteins for extracellular ADA (48). Ecto-ADA anchored on DC surface through A_2B_R and DPP4 on T cells compose a ternary complex and potentiates Th1-like cell activation and high production levels of Th1 cytokines such as IFN-γ, IL-6 and TNF-α, but without affecting Th2 cytokine production (49). Another study reported that A_2A_R can also compose similar ternary complex (50). Direct interaction of DPP4 on T cell with ADA or its activating antibody results in the recruitment of CD45 in lipid raft, forming a signaling complex. The DPP4/CD45 complex then enhances the phosphorylation of downstream CD3-ζ, p56lck, and zap-70, providing co-stimulatory signal for T cell activation (51, 52). Additionally, DPP4 on T cells interacts may also directly interact with caveolin-1 on APCs. This interaction triggers CARMA1-mediated nuclear factor (NF)-κB activation and its downstream signaling, resulting in T cells proliferation in a TCR/CD3-dependent manner (53). Co-stimulatory blockade of DPP4 by DPP4 deletion or pharmacological inhibition results in a significant reduction of IL-17 and IL-21 cytokines from CD4+ T cells, suggesting a critical role of DPP4 in Th17 activation (23).

### B Cells

DPP4 is expressed at a very lower level (less than 2%) in unstimulated CD20+ B cells, but significantly upregulated to around 50% through specific stimuli [including St. aureus and pokeweed mitogen(PWM)]. This result suggests a potential involvement of DPP4 in B cells activation (28). Incubation with DPP4 inhibitors suppress DNA synthesis and IgM secretion by B cells in a dose-dependent manner (28). In supporting this, DPP4 knockout mice, displayed a markedly decreased production of IgG after immunization by PWM. The decreased production of IL-4 and IL-2, delayed IFN-γ secretion in sera possibly contribute to the decreased Immunoglobulin production and impaired immunoglobulin isotype switching to IgG1, IgG2a and IgE (27). DPP4 is expressed in some B cell chronic lymphocytic leukemia cell linage and associated with the prognosis (54). In conclusion, DPP4 may serve as an activation marker for B cells, although its exact role in B cell biology is still not completely understood.

### NKs

A serial of studies revealed that DPP4 might be a characteristic surface marker of NKs. In normal condition, NKs express DPP4 at a low frequency, but is dramatically increased to 30% after IL-2 stimulation (31, 55, 56). Researchers utilized peripheral blood CD16^+^CD56^+^ NK cell to confirm that DPP4 could be induced by IL-2, IL-15 or IL-12, and regarded as a potential activation marker for NK cells (57). DPP4 is able to induce protein tyrosine phosphorylation and elicit a CD16-dependent lytic response in NKs (58). In addition, DPP4 was reported to sustain NK cytotoxicity against lung cancer (6, 32).

### DCs

The expression of DPP4 on DC was first detected on a restricted subpopulation in afferent lymph nodes (29).Flow cytometry analysis further suggested DPP4 was expressed at high levels on cDCs (59–61). Moreover, the expression of DPP4 on DCs was higher in visceral adipose tissue (VAT) from obese mice and humans compared with lean controls (30). During *in vitro* DCs differentiation, a significant increase in DPP4 expression was detected, suggesting a link between DPP4 expression and maturation of DCs (30). DPP4 positive DCs contribute to adaptive immunity, especially Th1-like responses. On one hand, DPP4 expressed on DC confers the ability to modify the macrophage-derived chemokines, which may attract Th1 cells (29). On the other hand, DPP4 modulates adenosine concentration and inflammation in the microenvironment of VAT by binding to ADA (30).

### Macrophages

DPP4 expression is also detected on macrophages from visceral adipose tissue in both high fat diet-induced and genetically obese mice, and the expression increased with functional maturation of macrophages (30). A long-term DPP4 inhibition reduced inflammation in VAT *via* downregulating proinflammatory genes in adipose tissue macrophages, and prevented monocyte migration and actin polymerization (33). Likewise, DPP4 inhibitions by alogliptin (62) and shRNA silencing (63) suppressed macrophages infiltration and accumulation. Like in DCs, DPP4 expression on macrophage was able to promote T-cell proliferation *via* modulating adenosine concentrations in micro-environment (30).

### Enzymatic Degradation of Immunoregulating Small Molecules

As discussed above, DPP4 is able to process a number of cytokines and chemokines by cleavage of N-terminal dipeptides.

Stromal cell-derived factor-1 (SDF-1), also as known as CXCL12, is a chemoattractant for T cells, hematopoietic progenitor cells, and adipose-derived regenerative cells (64–66). SDF-1 can be proteolytic cleaved by DPP4 and converted into CXCL12(3-68) (67). CXCL12(3-68) failed to induce CXCR4-mediated β-arrestin recruitment and downstream activation of IP3, Akt or ERK1/2, and thus losing its chemoattractant properties to lymphocytes. Co-administration of DPP4 inhibitor sitagliptin significantly enhanced the ability of intact SDF-1, but not CXCL12(3-68), to induce intra-articular lymphocyte infiltration (67). In addition to inactivating SDF-1, DPP4 also regulates SDF-1-mediated lymphocyte migration through direct interaction with its receptor CXCR4. DPP4 binds to CXCR4 on T lymphocytes and SDF-1 is able to induce the internalization of CXCR4/DPP4 complex. Interestingly, internalized CXCR4 is rapidly recycled back to the plasma membrane while DPP4 is clustered in intracellular vesicles, suggesting a self-regulatory mechanism of SDF-1 in reducing DPP4-dependendt inactivation (68). Other chemokines identified to be truncated by DPP4 include IP10, MIP, MIG, I-TAC, MDC, RANTES, etc. (**Table 2**).

**Table 2:** Summary of DPP4 substrates.

Peptide	N-terminus	Species	Functional change after truncation	Physiological Function	References
*Chemokines and cytokines*					
CXCL9(Mig)	**TP**↓VVRK…	Human	Reduce activity	Lymphocyte chemotaxis	(69)
CXCL10 (IP-10)	**VP**↓LSRT	Human	Reduce activity	Lymphocyte chemotaxis	(69, 70)
CXCL11(I-TAC)	**FP**↓MFKR	Human	Reduce activity	Lymphocyte chemotaxis	(69, 71)
CXCL12(SDF-1a)	**KP**↓VSLS…	Human	Reduce activity	Lymphocyte chemotaxis	(67, 69)
CCL2(MCP-1)	**QP**↓DAV…	Human	Increase activity	Angiogenesis	(72)
CCL3(MIP-1α)	**AP**↓YGA…	Murine	Increase activity	Monocyte chemotaxis	(34)
CCL3L1(LD78β)	**AP**↓LAAD…	Human	Affinity alteration	Monocyte chemotaxis	(73, 74)
CCL5(RANTES)	**SP**↓YSSD…	Human	Reduce activity	Macrophage CSF	(75)
CCL11(Eotaxin)	**GP**↓ASV…	Human	Reduce activity	Eosinophil chemotaxis	(69, 76)
CCL22(MDC)	**GP**↓YG↓AN	Human	Reduce activity	Lymphocyte chemotaxis	(77, 78)
*Cytokines*					
IL-3	**AP**↓MTQ…	Human	Reduce activity	Cell proliferation	(79)
GM-CSF	**AP**↓ARS…	Human	Reduce activity	Cell proliferation	(79)
G-CSF	**AT**↓PLG…	Human	Reduce activity	Cell proliferation	(79)
EPO	**AT**↓PLG…	Human	Reduce activity	Cell proliferation	(79)
FGF2	**PA**↓LPE…	Human	loss nuclear localization signal	Inhibition *in vitro* lead to metastatic potential of prostate cancer cells	(80, 81)
*Incretin hormones*					
GLP-1	**HA**↓EGTFTSD-	Human	Inactivation	Postprandial insulin response	(82)
GLP-2	**HA**↓DG↓SF…	Human	Inactivation	Glucose control	(82)
GIP	**YA**↓EGTF…	Human	Inactivation	Postprandial insulin response	(82)
PACAP	**HS**↓EG↓IF…	Human	Inactivation	Neural regulation of islet	(82)
GRP	**VP**↓LP↓AG…	Human	Inactivation	Neural regulation of islet	(82)
*Neuropeptides*					
Neuropeptide Y	**YP**↓SKPDNPG	Human	Affinity alteration	inhibit exocrine pancreas function, feeding	(83)
Peptide YY	**YP**↓IKPEAPG	Human	Affinity alteration	Multiple function in renal, digestive system and food intake	(84)

Other cytokines degraded by DPP4 include fibroblast growth factor 2 (FGF2), IL-3, granulocyte-macrophage colony stimulating factor (GM-CSF), granulocyte colony stimulating factor (G-CSF), IL-3, and erythropoietin (EPO) (79–81). Many interleukin family members such as IL-2/-5/-10/-13/-17/-22/-23/-27/-28 also possess potential cleavage site of DPP4 (80). However, further biochemical and biological studies are needed to identify whether the putative DPP4 truncation sites are true truncation sites for those peptides.

## The Role of DPP4 in Fibrosis

Fibroblasts are spindle-shaped cells responsible for the synthesis of extracellular matrix and collagen in connective tissue (85, 86). Although fibroblasts are not conventional immune cells, they play an important role in immune regulation and autoimmune disease. Fibroblasts may also act as an antigen present cell to promote proliferation, activation, and recruitment of adaptive immune cells *via* both direct cell-to-cell interaction and secretion of cytokines (87, 88). The expression of DPP4 was found to at a high level in capsule fibroblasts compared to medulla fibroblasts in the thymus of mice, suggesting that DPP4 may serve as a segregate marker to distinguish different fibroblast subsets (35). Fibroblasts are center actor in fibrosis and wound healing (16, 89). SFRP2/DPP4 define a major fibroblast population in human skin (90). The expression of DPP4 allows isolation of a fibrogenic, scar forming lineage and inhibition of DPP4 reduces cutaneous scarring during wound healing (16). Another study suggested that the expression of DPP4 in the skin fibroblast was upregulated in patients with systemic sclerosis compared with that of healthy individuals. DPP4 regulates TGF-β-induced fibroblast activation in skin fibrosis and DPP4 characterizes an activated population of fibroblasts characterized by the expression of collagen and myofibroblast markers. Genetic deletion or pharmacologic inhibition of DPP4 in fibroblasts suppressed their proliferation, migration, and collagen production (91). Hui-Chun Ku et al. reported that DPP4 activated dermal fibroblasts *via* PAR2 and downstream NF-κB/SMAD signaling (92). Expression of DPP4 was increased in the thickening peritoneum of chlorhexidine gluconate-induced peritoneal fibrosis model of rats. Increased expression of DPP4 in diabetes was found to promote epithelial-mesenchymal transition and peritoneal fibrosis, which could be relieved by pharmacological or genetic inactivation of DPP4 (93). Moreover, DPP4 binding to extracellular matrix proteins (such as collagen and fibronectin) and extracellular matrix degrading enzymes (such as plasminogen and streptokinase) probably contributes to cells spreading and metastasis (5, 94, 95). Streptokinase, plasminogen and its metabolite plasmin bind to cysteine-rich region of DPP4, resulting in a rapid increase of intracellular Ca^2+^ response and subsequent activation of fibroblast (5, 96). The binding of DPP4 with plasminogen also regulates the homeostasis of extracellular matrix by promoting the secretion of matrix metalloproteinases and conversion of plasminogen to plasmin (97). In addition, production of plasmin is able to degrade BP180, an autoantigen found in autoimmune skin disease bullous pemphigoid. Therefore, DPP4 may be involved in the maintaining of BP180 immunotolerance and the prevention of BP autoantibody production (98).

## The Role of DPP4 in Autoimmune Disease

### DPP4 in Rheumatoid Arthritis

Rheumatoid arthritis (RA) is a chronic progressive autoimmune disease causing pain, swelling, stiffness and loss of function of joints. Although the exact cause and pathogenesis are unclear, it’s a consensus that T cell activation plays an important role in the initiation and maintaining of inflammation in RA (99). Various factors involved in T cell activation, including CD28, CD40, CTLA4, ILRA/IL2, IL-21, have been found to increase in RA (100). As an activation marker of T cells, DPP4 has received increasing attention in RA. Serum DPP4 concentration is significantly decreased in patients with RA compared to the control group (101), and this decrease is inversely correlated to disease activity (102). However, DPP4 expression on the surface of circulating lymphocytes and monocytes showed no significant differences between early RA patients and healthy controls (102, 103). Another study suggested that DPP4 expression on peripheral blood CD4+ T cells was higher in active RA compared to inactive RA and controls. However, its expression on synovial fluid T cells from RA was lower than that from osteoarthritis patients (104). The decrease of DPP4 activity also occurs in synovial fluids, fluid mononuclear cells (FMNC) and synovial membrane in RA compared with osteoarthritis (105–107). Interestingly, Anti-DPP4 autoantibodies have been observed in RA patients and may be used as a biomarker for early diagnosis of RA (108), which suggests that DPP4 may contributes to the immunopathology of RA.

SDF-1, a substrate of DPP4, is thought to play a central role in inflammatory cell recruitment *via* interacting with its receptor CXCR4. In murine model of antigen-induced arthritis, DPP4 deficiency resulted in preservation of serum active SDF-1 and enhanced infiltration of CXCR4-positive inflammatory cells in arthritic joints (109). The plasma level of DPP4 was lower than that in osteoarthritis and negatively correlated with plasma inflammation marker C-reactive protein (109). The level of SDF-1 was inversely correlated with the number of DPP4+ T cells in synovial fluids from patients with RA (105). Since the synovial level of SDF-1 was strongly correlated with the disease activity score (DAS28 CRP) and inflammation markers (serum C-reactive protein and IL-6) (109, 110), the decrease of synovial DPP4 in RA may lead to synovial inflammation *via* SDF-1/CXCR4 axis. These results indicate a critical role of DPP4/SDF1/CXCR4 in synovial inflammation in RA.

A recent study indicates that exogenous DPP4 or overexpression of DPP4 in synovial fibroblasts reduced the production of proinflammatory cytokines such as IL-1β, IL-6 and IL-3 from fibroblasts (111). Bone erosion is a severe consequence in RA progression. In a recent study, DPP4 was found to be highly expressed in osteoclast and its expression was suppressed by an anti-resorptive agent denosumab, suggesting that osteoclast-derived DPP4 may be an important link between energy metabolism and bone remodeling (112). It is shown that the invasion of synovial fibroblasts into cartilage was enhanced by the inhibition of DPP4 in a mouse model of RA (113). However, in a streptozotocin-induced diabetic model of rat, DPP4 inhibitor was shown to attenuate the bone loss and improve mechanical bone strength, probably through reducing CTX-I-dependent bone resorption (114).

Clinical observation on patients prescribed with DPP4 inhibitors may offer critical information on role of DPP4 enzymatic activities in RA. DPP4 inhibitors-associated newly onset RA cases have been reported by several groups (115–117). However, larger-scale population studies failed to identify an association between RA and DPP4i utilization compared to other antidiabetic therapies (118–121). Future randomized controlled trials are required to investigate the exact effects of DPP4 enzymatic inhibition on the development of RA.

### DPP4 in Systemic Lupus Erythematosus

Systemic lupus erythematosus (SLE) is a chronic multi-system autoimmune disease with many patients presenting with a characteristic butterfly rash (erythematous rash over the cheeks and bridge of the nose). Since most patients present high titers of autoreactive antibodies and disease activity is correlated with autoantibody titers, SLE used to be considered as an adaptive immune system disorder (122).

Serum DPP4 activities were significantly decreased in mice with lupus erythematosus-like syndrome compared to healthy mice, indicating a potential involvement of DPP4 in the pathogenesis of SLE (4). Likewise, clinical evidences demonstrate lower levels of DPP4 in the serum and peripheral blood mononuclear cells of SLE patients compared to health controls (4, 123, 124). Wong et al. reported that surface expression of DPP4 on CD4+ T cell and invariant natural killer T (iNKT) lymphocytes were reduced in SLE patients, accompanied with reduced circulating iNKT and elevated Th1 response (125). DPP4 mRNA expression analysis *via* qPCR showed 3.6-fold higher in SLE group than in the healthy control group, whereas no significant correlation with disease activity (50).

Recent population-cohort studies from Taiwan and Korea showed that DPP4 inhibitors was associated with a reduced risk of SLE (126, 127). However, high-level clinical evidence on the relation between DPP4 inhibition and SLE is limited.

### DPP4 in Systemic Sclerosis

Systemic Sclerosis (SSc), also known as scleroderma, is another severe autoimmune disease characterized by diffuse cutaneous and visceral fibrosis (128). Early research reported an increase in both absolute number and percentage of peripheral blood CD4^+^DPP4^+^T in scleroderma patients, and the levels of DPP4 expression in T cells correlated with disease activity (129). Circulating soluble DPP4 was also significantly decreased in SSc patients, compared to controls (101). Compared to limited cutaneous SSc, the soluble DPP4 levels further reduced in diffuse cutaneous SSc (130), which supports the hypothesis that DPP4 activity is associated with fibrosis progress in SSc.

Myofibroblasts are main collagen-producing cells in tissue fibrosis (131). In SSc skin, myofibroblasts contribute to tissue tension and skin/joint contractures (132, 133). As discussed above, DPP4 is critical for the activation of fibroblast. DPP4-positive fibroblasts express a high level of profibrotic genes including collagen-1, collagen-3, and fibronectin (134). Additionally, stimulation with recombinant human DPP4 promotes the production of fibronectin in lung fibroblast, suggesting a role of DPP4 in fibroblast activation and tissue remodeling (135). A recent study discovered that DPP4-expressing fibroblasts are responsible for collagen deposition in dermal scars and inhibition of DPP4 reduced scar formation in a mouse model of wound healing (16). The following studies demonstrated that SFRP2/DPP4 fibroblast subpopulation is the progenitor of fibrogenic fibroblasts in SSc skin (136) and DPP4 activated NF-κB and SMAD signaling through PAR2, leading to the activation of dermal fibroblasts (92).

Utilization of DPP4 inhibitors sitagliptin and vildagliptin in murine model of bleomycin-induced skin fibrosis showed a marked anti-fibrotic effect as evidenced by ameliorated dermal thickness, hydroxyproline content, and accumulation of myofibroblasts through suppressing TGF-β-induced ERK signaling pathway (91). Vildagliptin also effectively attenuated fibrosis and inflammation in bleomycin-induced lung fibrosis (137). Although animal studies have suggested a prospective application of DPP4 inhibitors in SSc, there are limited clinical trials investigating the role of DPP4 inhibitors in patients with SSc.

### DPP4 in Inflammatory Bowel Disease

Inflammatory bowel disease (IBD) is a typical autoimmune-mediated digestive system disease including Crohn’s disease (CD) and ulcerative colitis (UC). Although there are differences between CD and UC, they both are characterized by chronic relapsing progressive process and multi-organ involvement in later stage. The serum DPP4 level and enzymatic activity in patients with IBD were decreased significantly compared to healthy control or patients with remissive conditions, and the extent of decrease was correlated with disease activity (138–141). In addition, responders to treatments had a higher serum DPP4 level compared to nonresponders (139). DPP4 activity in the fecal was found to reduce in active UC patients, but increase in CD patients, when compared to remitters (142). These results suggest that DPP4 activity is a potential biomarker for monitoring IBD activity and therapeutic response (143). A model of TNBS-induced colitis in DPP4^-/-^ mice showed higher serum levels of neuropeptide Y (NPY), vasoactive intestinal peptide (VIP) and IL-6, which are all substrates of DPP4, compared to C57BL/6 mice. The levels of VIP and IL-6 in the colon and brain tissues were also increased in DPP4^-/-^ mice during the acute inflammation phase of colitis. IL-10 in the brain was found to reduce in wild-type mice, but increase in DPP4^-/-^ mice, suggesting a potential role of DPP4 in regulating neuroimmune response in colitis development (144). In dextran sulfate sodium (DSS)-induced colitis, DPP4^-/-^ mice showed increased CD8+ T cells and NKT cells in the spleen, as well as increased macrophage infiltration and enhanced expression of NF-κB p65 subunit in colon mucosa (145),. Additionally, GLP-1 and GLP-2 promote the repair of injured intestinal epithelia and regulate T cell differentiation and functions, and those functions are inhibited by DPP4 enzymatic cleavage (146, 147). The protective effect of DPP4 inhibitors on IBD has been observed in animal studies. DSS-induced colitis in mice was improved by administration of DPP4 inhibitor ER-319711, as demonstrated by less colon shortening and weight loss (148). Likewise, anagliptin treatment also facilitates the restoration of intestinal mucosal damage in DSS-induced colitis in C57BL/6 mice (149). However, clinical evidence about the effect of DPP4 inhibitor on IBD is limited and inconsistent. A cross-sectional study observed an increased risk of several autoimmune diseases including Crohn’s disease, Hashimoto’s thyroiditis, and Psoriasis in patients on DPP4 inhibition therapy (150). Abrahami et al. also reported an association between the use of DPP4 inhibitor and increased risk of IBD (hazard ratio 1.75, 95% confidence interval 1.22 to 2.49) (151). Nevertheless, another real-world investigation of 895,747 patients on either DPP4 inhibition therapy or other anti-diabetic treatment suggested that the use of DPP4 inhibitors was not associated with increased IBD risk (152). A meta-analysis involving 16 individual studies also reported a non-significant increase in the risk of IBD after exposure to DPP4 inhibitor when using a random-effects model (Relative risk 1.52; 95% confidence interval 0.72 to 3.24). However, this finding was driven by the inclusion of a large study and further surveillance on this effect is warranted (153).

### DPP4 in Autoimmune Diabetes

Type 1 diabetes mellitus (T1DM), also known as autoimmune diabetes, is characterized by immune-mediated destruction of pancreatic β cells and insufficiency of insulin secretion in early ages (154). Serum DPP4 activity was found to increase in patients with T1DM and the elevation is correlated with duration of diabetes (155–157). Although DPP4i has been used as either mono-therapy or combined therapy for T2DM over a decade, there is limited evidence supporting its application in T1DM. The effect of DPP4 inhibition on lowering HbA1c in patients with T1DM was not consistent in clinical trials (158–161).

Despite inconsistent findings in clinical investigations, the role of DPP4 in T1DM progression and inflammatory process has received sufficient attention in preclinical studies. Imbalanced Th1/Th2 and Th17/Treg responses are important features of T1DM. DPP4 inhibition leads to decreased Th1 response, increase of Th2 cytokines, and restoration of Treg/Th17 imbalance (98). GLP-1, a substrate of DPP4, was reported to possess anti-inflammatory property in pancreas and adipose tissue by reducing the production of inflammatory cytokines and infiltration of immune cells (162). Therefore, the inhibition of DPP4 contribute to anti-inflammatory process in pancreatic. In NOD mice trial treated with DPP4 inhibitor MK0431, the survival of islet graft was prolonged, accompanied by a decreased migration of CD4^+^ T cells into pancreas through a pathway involving cAMP/PKA/Rac1 activation (163). In another T1DM mouse model induced by low-dose streptozotocin, sitagliptin not only reduced blood glucose level, but also improved inflammation in pancreas by increasing CD4^+^CD25^hi^Foxp3^+^ T cells and reducing inflammatory cells (such as CD11b^+^ cells and CD4^+^CD26^+^ T cells) (164). Therefore, preclinical studies indicate that DPP4 inhibition may improve T1DM through mechanisms involving both incretin effect and anti-inflammatory action.

### DPP4 in Other Autoimmune Diseases

In addition to diseases mentioned above, involvement of DPP4 in other autoimmune diseases are displayed in **Table 3**. However, given the lower incidence of certain autoimmune disease, limited evidences are accessible. Case reports and retrospective studies of DPP4i utilization in T2DM patients demonstrated an association between the use of DPP4i and bullous pemphigoid, a severe autoimmune skin disease (18, 173–177). Importantly, DPP4i-induced bullous pemphigoid does not remit fully after withdrawal of DPP4i, suggesting that DPP4i induces and aggravate the process of bullous pemphigoid rather than a reversible side effect of DPP4i (18). Although the exact mechanisms underlying DPP4i-associated bullous pemphigoid is currently unclear, the breakdown of immunotolerance of BP180, the major autoantigen of bullous pemphigoid, might be a key reason. As mentioned above, DPP4 is involved in the immunotolerance of BP180 by regulating the conversion of plasminogen to plasmin that is responsible for the degradation of BP180 (98). A case-control study found that salivary DPP4 activity was increased in the patients with Sjögren’s syndrome (SS), and there was a positive correlation between DPP4 activity and MMP9 level (166). A serial of studies reported that DPP4 is associated with chondrocyte physiology and inhibition of DPP4 suppresses the degradation of ECM, which is considered to help the amelioration of osteoarthritis (169, 178, 179). In addition, DPP4 inhibition was also reported to improve psoriasis (172), probably by inhibiting T cell activation (171). Collectively, further animal and clinical studies are required to identify the exact role of DPP4 in the development of autoimmune diseases.

**Table 3:** The role of DPP4 in autoimmune diseases.

	Expression	Mechanisms	Effect of DPP4 inhibitor on disease phenotype
**RA**	Decreased in serum (101)	Limit the recruitment of inflammatory cells (109, 110) Involved in bone erosion (114)	Inconsistent: several case reports indicate DPP4 inhibitors induce RA (117, 126), some studies reported no association or reduced risk of RA (120)
**SLE**	Decreased in serum (4, 123, 124)	Evidence limited	Reduce risk (126)
**SSc**	Increased in T cells (129)	Indication of activated fibroblast	Limited evidence in humans; Animal study shows DPP4 inhibition meliorates fibrosis *in vivo (* 91, 137)
**IBD**	Decreased serum activity (140, 165)	Regulate neuroimmune response (145) Impair tissue recovery by inactivation of GLP-2 (147)	Biomarker for treatment response (143) DPP4i promote prognosis in animals (149) Increased IBD risks (150, 151), or no impact (152, 153)
**SS**	Increased in saliva (166)	Regulate expression of MMP9 (166)	Evidence limited
**OA**	No significant alteration (167)	Upregulate AGE-induced MMPs (168) Promote bone formation by inactivating GLP-1 (168)	Improve ECM loss (168, 169)
**Psoriasis**	Increased at mRNA level in lesion (170)	inhibit T cell activation (171)	Improve psoriasis severity (172)

## Discussion

This review discusses the role of DPP4 in immune system and its role in pathogenesis of different autoimmune diseases. Apparently, the expression of DPP4 is significantly affected in different autoimmune conditions. However, DPP4 is not a specific marker of any autoimmune disease, as its ubiquitous expression limits the potential of DPP4 as a precise biomarker. In addition to autoimmune disease, immune cell-derived DPP4 may also other disease conditions such as type 2 diabetes. Studies have demonstrated that immune cells, especially circulating CD4+ T helper cells, are important source of plasma DPP4 activity which is responsible for postprandial glucose intolerance in patients with type 2 diabetes (180, 181). In patients with type 2 diabetes, kallikrein-related peptidase 5, an enzyme responsible for the shedding of DPP4 from cell membrane, was induced in CD4+ T cells, suggesting that immune cell-derived DPP4 is responsible for reduced incretin effect in diabetes patients (180). Experimental and clinical investigations suggest that DPP4 may play a dual role in the pathogenesis of autoimmune diseases and the inhibition of DPP4 results distinct outcome in different disease condition. A possible reason is that DPP4, as a moonlighting protein, possesses diverse functions including enzymatic degradation of various substrates and enzymatic independent interaction with many ligands. In addition, while DPP4 is widely expressed in many types of cells, DPP4 expression in different cell population may also have distinct functions. To dissect the exact role of DPP4 in autoimmune diseases, future efforts may focus on the role of DPP4 in different types of cells, the temporal and spatial characteristics of DPP4 expression (especially in different stages of disease), unrecognized ligands for DPP4, and strategies targeting the non-enzymatic activity of DPP4. Taken together, DPP4 is a promising target of autoimmune diseases although its exact mechanisms in these conditions remain elucidated.

## Author Contributions

JH performed literature search. JH, XXL, YW, XLL, and SG drafted the manuscript. LD, XR, and JZ revised the manuscript. All author reviewed the manuscript. All authors contributed to the article and approved the submitted version.

## Funding

This work was supported by the National Natural Science Foundation of China (grant numbers 81974254, 31870906, 82170470, and 81670431).

## Conflict of Interest

The authors declare that the research was conducted in the absence of any commercial or financial relationships that could be construed as a potential conflict of interest.

## Publisher’s Note

All claims expressed in this article are solely those of the authors and do not necessarily represent those of their affiliated organizations, or those of the publisher, the editors and the reviewers. Any product that may be evaluated in this article, or claim that may be made by its manufacturer, is not guaranteed or endorsed by the publisher.

## References

[1] Hopsu-Havu VK Glenner GG. A New Dipeptide Naphthylamidase Hydrolyzing Glycyl-Prolyl-Beta-Naphthylamide. Histochemie (1966) 7(3):197–201. doi: 10.1007/BF00577838 5959122

[2] De Meester I Korom S Van Damme J Scharpé S. CD26, Let it Cut or Cut it Down. Immunol Today (1999) 20(8):367–75. doi: 10.1016/S0167-5699(99)01486-3 10431157

[3] Thul PJ Åkesson L Wiking M Mahdessian D Geladaki A Ait Blal H. A Subcellular Map of the Human Proteome. Science (2017) 356(6340):eaal3321. doi: 10.1126/science.aal3321 28495876

[4] Hagihara M Ohhashi M Nagatsu T. Activities of Dipeptidyl Peptidase II and Dipeptidyl Peptidase IV in Mice With Lupus Erythematosus-Like Syndrome and in Patients With Lupus Erythematosus and Rheumatoid Arthritis. Clin Chem (1987) 33(8):1463–5. doi: 10.1093/clinchem/33.8.1463 2886236

[5] Klemann C Wagner L Stephan M von Hörsten S. Cut to the Chase: A Review of CD26/dipeptidyl Peptidase-4’s (DPP4) Entanglement in the Immune System. Clin Exp Immunol (2016) 185(1):1–21. doi: 10.1111/cei.12781 26919392 PMC4908298

[6] Vahl TP Paty BW Fuller BD Prigeon RL D'Alessio DA. Effects of GLP-1-(7-36)NH2, GLP-1-(7-37), and GLP-1- (9-36)NH2 on Intravenous Glucose Tolerance and Glucose-Induced Insulin Secretion in Healthy Humans. J Clin Endocrinol Metab (2003) 88(4):1772–9. doi: 10.1210/jc.2002-021479 12679472

[7] Lambeir A-M Durinx C Scharpé S De Meester I. Dipeptidyl-Peptidase IV From Bench to Bedside: An Update on Structural Properties, Functions, and Clinical Aspects of the Enzyme DPP IV. Crit Rev Clin Lab Sci (2003) 40(3):209–94. doi: 10.1080/713609354 12892317

[8] Raj VS Mou H Smits SL Dekkers DHW Müller MA Dijkman R. Dipeptidyl Peptidase 4 is a Functional Receptor for the Emerging Human Coronavirus-EMC. Nature (2013) 495(7440):251–4. doi: 10.1038/nature12005 PMC7095326 23486063

[9] Wang N Shi X Jiang L Zhang S Wang D Tong P. Structure of MERS-CoV Spike Receptor-Binding Domain Complexed With Human Receptor DPP4. Cell Res (2013) 23(8):986–93. doi: 10.1038/cr.2013.92 PMC3731569 23835475

[10] Lu G Hu Y Wang Q Qi J Gao F Li Y. Molecular Basis of Binding Between Novel Human Coronavirus MERS-CoV and its Receptor CD26. Nature (2013) 500(7461):227–31. doi: 10.1038/nature12328 PMC7095341 23831647

[11] Meyerholz DK Lambertz AM McCray PB Jr. Dipeptidyl Peptidase 4 Distribution in the Human Respiratory Tract: Implications for the Middle East Respiratory Syndrome. Am J Pathol (2016) 186(1):78–86. doi: 10.1016/j.ajpath.2015.09.014 26597880 PMC4715219

[12] Callebaut C Krust B Jacotot E Hovanessian AG. T Cell Activation Antigen, CD26, as a Cofactor for Entry of HIV in CD4+ Cells. Science (1993) 262(5142):2045–50. doi: 10.1126/science.7903479 7903479

[13] Alkhatib G Combadiere C Broder CC Feng Y Kennedy PE Murphy PM. CC CKR5: A RANTES, MIP-1alpha, MIP-1beta Receptor as a Fusion Cofactor for Macrophage-Tropic HIV-1. Science (1996) 272(5270):1955–8. doi: 10.1126/science.272.5270.1955 8658171

[14] Deng H Liu R Ellmeier W Choe S Unutmaz D Burkhart M. Identification of a Major Co-Receptor for Primary Isolates of HIV-1. Nature (1996) 381(6584):661–6. doi: 10.1038/381661a0 8649511

[15] Wu L Paxton WA Kassam N Ruffing N Rottman JB Sullivan N. CCR5 Levels and Expression Pattern Correlate With Infectability by Macrophage-Tropic HIV-1, *In Vitro* . J Exp Med (1997) 185(9):1681–91. doi: 10.1084/jem.185.9.1681 PMC2196298 9151905

[16] Rinkevich Y Walmsley GG Hu MS Maan ZN Newman AM Drukker M. Skin Fibrosis. Identification and Isolation of a Dermal Lineage With Intrinsic Fibrogenic Potential. Science (2015) 348(6232):aaa2151. doi: 10.1126/science.aaa2151 25883361 PMC5088503

[17] Merrick D Sakers A Irgebay Z Okada C Calvert C Morley MP. Identification of a Mesenchymal Progenitor Cell Hierarchy in Adipose Tissue. Science (2019) 364(6438):eaav2501. doi: 10.1126/science.aav2501 31023895 PMC6816238

[18] Roy A Sahoo J Narayanan N Merugu C Kamalanathan S Naik D. Dipeptidyl Peptidase-4 Inhibitor-Induced Autoimmune Diseases: Current Evidence. World J Diabetes (2021) 12(9):1426–41. doi: 10.4239/wjd.v12.i9.1426 PMC8472501 34630898

[19] Zhao Y Yang L Wang X Zhou Z. The New Insights From DPP-4 Inhibitors: Their Potential Immune Modulatory Function in Autoimmune Diabetes. Diabetes Metab Res Rev (2014) 30(8):646–53. doi: 10.1002/dmrr.2530 24446278

[20] Willheim M Ebner C Baier K Kern W Schrattbauer K Thien R. Cell Surface Characterization of T Lymphocytes and Allergen-Specific T Cell Clones: Correlation of CD26 Expression With T(H1) Subsets. J Allergy Clin Immunol (1997) 100(3):348–55. doi: 10.1016/S0091-6749(97)70248-3 9314347

[21] Lun SWM Wong CK Ko FWS Hui DSC Lam CWK. Increased Expression of Plasma and CD4+ T Lymphocyte Costimulatory Molecule CD26 in Adult Patients With Allergic Asthma. J Clin Immunol (2007) 27(4):430–7. doi: 10.1007/s10875-007-9093-z 17525828

[22] Bengsch B Seigel B Flecken T Wolanski J Blum HE Thimme R. Human Th17 Cells Express High Levels of Enzymatically Active Dipeptidylpeptidase IV (Cd26). J Immunol (2012) 188(11):5438–47. doi: 10.4049/jimmunol.1103801 22539793

[23] Yamada Y Jang JH De Meester I Baerts L Vliegen G Inci I. CD26 Costimulatory Blockade Improves Lung Allograft Rejection and is Associated With Enhanced Interleukin-10 Expression. J Heart Lung Transplant (2016) 35(4):508–17. doi: 10.1016/j.healun.2015.11.002 26755203

[24] Salgado FJ Pérez-Díaz A Villanueva NM Lamas O Arias P Nogueira M. CD26: A Negative Selection Marker for Human Treg Cells. Cytometry A (2012) 81(10):843–55. doi: 10.1002/cyto.a.22117 22949266

[25] Hatano R Ohnuma K Yamamoto J Dang NH Morimoto C. CD26-Mediated Co-Stimulation in Human CD8(+) T Cells Provokes Effector Function *via* Pro-Inflammatory Cytokine Production. Immunology (2013) 138(2):165–72. doi: 10.1111/imm.12028 PMC3575769 23113658

[26] Ibegbu CC Xu Y-X Fillos D Radziewicz H Grakoui A Kourtis AP. Differential Expression of CD26 on Virus-Specific CD8(+) T Cells During Active, Latent and Resolved Infection. Immunology (2009) 126(3):346–53. doi: 10.1111/j.1365-2567.2008.02899.x PMC2669815 18657205

[27] Yan S Marguet D Dobers J Reutter W Fan H. Deficiency of CD26 Results in a Change of Cytokine and Immunoglobulin Secretion After Stimulation by Pokeweed Mitogen. Eur J Immunol (2003) 33(6):1519–27. doi: 10.1002/eji.200323469 12778469

[28] Buhling F Junker U Reinhold D Neubert K Jäger L Ansorge S. Functional Role of CD26 on Human B Lymphocytes. Immunol Lett (1995) 45(1-2):47–51. doi: 10.1016/0165-2478(94)00230-O 7622187

[29] Gliddon DR Howard CJ. CD26 is Expressed on a Restricted Subpopulation of Dendritic Cells *In Vivo* . Eur J Immunol (2002) 32(5):1472–81. doi: 10.1002/1521-4141(200205)32:5<1472::AID-IMMU1472>3.0.CO;2-Q 11981836

[30] Zhong J Rao X Deiuliis J Braunstein Z Narula V Hazey J. A Potential Role for Dendritic Cell/Macrophage-Expressing DPP4 in Obesity-Induced Visceral Inflammation. Diabetes (2013) 62(1):149–57. doi: 10.2337/db12-0230 PMC3526020 22936179

[31] Buhling F Kunz D Reinhold D Ulmer AJ Ernst M Flad HD. Expression and Functional Role of Dipeptidyl Peptidase IV (CD26) on Human Natural Killer Cells. Nat Immun (1994) 13(5):270–9. 7833626

[32] Shingu K Helfritz A Zielinska-Skowronek M Meyer-Olson D Jacobs R Schmidt RE. CD26 Expression Determines Lung Metastasis in Mutant F344 Rats: Involvement of NK Cell Function and Soluble CD26. Cancer Immunol Immunother (2003) 52(9):546–54. doi: 10.1007/s00262-003-0392-9 PMC11034350 14627126

[33] Shah Z Kampfrath T Deiuliis JA Zhong J Pineda C Ying Z. Long-Term Dipeptidyl-Peptidase 4 Inhibition Reduces Atherosclerosis and Inflammation *via* Effects on Monocyte Recruitment and Chemotaxis. Circulation (2011) 124(21):2338–49. doi: 10.1161/CIRCULATIONAHA.111.041418 PMC4224594 22007077

[34] Zhuge F Ni Y Nagashimada M Nagata N Xu L Mukaida N. DPP-4 Inhibition by Linagliptin Attenuates Obesity-Related Inflammation and Insulin Resistance by Regulating M1/M2 Macrophage Polarization. Diabetes (2016) 65(10):2966–79. doi: 10.2337/db16-0317 27445264

[35] Nitta T Tsutsumi M Nitta S Muro R Suzuki EC Nakano K. Fibroblasts as a Source of Self-Antigens for Central Immune Tolerance. Nat Immunol (2020) 21(10):1172–80. doi: 10.1038/s41590-020-0756-8 32839611

[36] Gorrell MD Wickson J McCaughan GW. Expression of the Rat CD26 Antigen (Dipeptidyl Peptidase IV) on Subpopulations of Rat Lymphocytes. Cell Immunol (1991) 134(1):205–15. doi: 10.1016/0008-8749(91)90343-A 1672835

[37] Tanaka T. Cloning and Functional Expression of the T Cell Activation Antigen CD26. J Immunol (1993) 150(5):2090. 8094732

[38] Mandapathil M Hilldorfer B Szczepanski MJ Czystowska M Szajnik M Ren J. Generation and Accumulation of Immunosuppressive Adenosine by Human CD4+CD25highFOXP3+ Regulatory T Cells. J Biol Chem (2010) 285(10):7176–86. doi: 10.1074/jbc.M109.047423 PMC2844167 19858205

[39] Dang NH Torimoto Y Shimamura K Tanaka T Daley JF Schlossman SF. 1f7 (CD26): A Marker of Thymic Maturation Involved in the Differential Regulation of the CD3 and CD2 Pathways of Human Thymocyte Activation. J Immunol (1991) 147(9):2825–32. 1717577

[40] Klemann C Schade J Pabst R Leitner S Stiller J von Hörsten S. CD26/dipeptidyl Peptidase 4-Deficiency Alters Thymic Emigration Patterns and Leukcocyte Subsets in F344-Rats Age-Dependently. Clin Exp Immunol (2009) 155(2):357–65. doi: 10.1111/j.1365-2249.2008.03839.x PMC2675268 19055685

[41] Frerker N Raber K Bode F Skripuletz T Nave H Klemann C. Phenotyping of Congenic Dipeptidyl Peptidase 4 (DP4) Deficient Dark Agouti (DA) Rats Suggests Involvement of DP4 in Neuro-, Endocrine, and Immune Functions. Clin Chem Lab Med (2009) 47(3):275–87. doi: 10.1515/CCLM.2009.064 19327106

[42] SchÖn E Demuth HU Eichmann E Horst HJ Körner IJ Kopp J. Dipeptidyl Peptidase IV in Human T Lymphocytes. Impaired Induction of Interleukin 2 and Gamma Interferon Due to Specific Inhibition of Dipeptidyl Peptidase IV. Scand J Immunol (1989) 29(2):127–32. doi: 10.1111/j.1365-3083.1989.tb01108.x 2564215

[43] Reinhold D Bank U Bühling F Täger M Born I Faust J. Inhibitors of Dipeptidyl Peptidase IV (DP IV, CD26) Induces Secretion of Transforming Growth Factor-Beta 1 (TGF-Beta 1) in Stimulated Mouse Splenocytes and Thymocytes. Immunol Lett (1997) 58(1):29–35. doi: 10.1016/S0165-2478(97)02716-8 9436466

[44] Tanaka T Kameoka J Yaron A Schlossman SF Morimoto C. The Costimulatory Activity of the CD26 Antigen Requires Dipeptidyl Peptidase IV Enzymatic Activity. Proc Natl Acad Sci U S A (1993) 90(10):4586–90. doi: 10.1073/pnas.90.10.4586 PMC46557 7685106

[45] von Bonin A Huhn J Fleischer B. Dipeptidyl-Peptidase IV/CD26 on T Cells: Analysis of an Alternative T-Cell Activation Pathway. Immunol Rev (1998) 161:43–53. doi: 10.1111/j.1600-065X.1998.tb01570.x 9553763

[46] Banchereau J Steinman RM. Dendritic Cells and the Control of Immunity. Nature (1998) 392(6673):245–52. doi: 10.1038/32588 9521319

[47] Lanzavecchia A Sallusto F. Dynamics of T Lymphocyte Responses: Intermediates, Effectors, and Memory Cells. Science (2000) 290(5489):92–7. doi: 10.1126/science.290.5489.92 11021806

[48] Herrera C Casadó V Ciruela F Schofield P Mallol J Lluis C. Adenosine A2B Receptors Behave as an Alternative Anchoring Protein for Cell Surface Adenosine Deaminase in Lymphocytes and Cultured Cells. Mol Pharmacol (2001) 59(1):127–34. doi: 10.1124/mol.59.1.127 11125033

[49] Pacheco R Martinez-Navio JM Lejeune M Climent N Oliva H Gatell JM Gallart T. CD26, Adenosine Deaminase, and Adenosine Receptors Mediate Costimulatory Signals in the Immunological Synapse. Proc Natl Acad Sci U S A (2005) 102(27):9583–8. doi: 10.1073/pnas.0501050102 PMC1172240 15983379

[50] Valizadeh M Ahmadzadeh A Behzadi M Yeganeh F. CD26 mRNA Expression in Systemic Lupus Erythematosus. Rheumatol Res (2018) 3(2):77–82. doi: 10.22631/rr.2018.69997.1045

[51] Ishii T Ohnuma K Murakami A Takasawa N Kobayashi S Dang NH. CD26-Mediated Signaling for T Cell Activation Occurs in Lipid Rafts Through its Association With CD45RO. Proc Natl Acad Sci U S A (2001) 98(21):12138–43. doi: 10.1073/pnas.211439098 PMC59781 11593028

[52] Torimoto Y Dang NH Vivier E Tanaka T Schlossman SF Morimoto C. Coassociation of CD26 (Dipeptidyl Peptidase IV) With CD45 on the Surface of Human T Lymphocytes. J Immunol (1991) 147(8):2514–7. 1680916

[53] Ohnuma K Uchiyama M Yamochi T Nishibashi K Hosono O Takahashi N. Caveolin-1 Triggers T-Cell Activation *via* CD26 in Association With CARMA1. J Biol Chem (2007) 282(13):10117–31. doi: 10.1074/jbc.M609157200 17287217

[54] Matuszak M Lewandowski K Czyż A Kiernicka-Parulska J Przybyłowicz-Chalecka A Jarmuż-Szymczak M. The Prognostic Significance of Surface Dipeptidylpeptidase IV (CD26) Expression in B-Cell Chronic Lymphocytic Leukemia. Leuk Res (2016) 47:166–71. doi: 10.1016/j.leukres.2016.06.002 27376546

[55] Biuling F Tonevitski AG Kiuster U Anzorge S. Study of Dipeptidyl Peptidase IV as a Surface Marker of Human Natural Killer Cells. Biull Eksp Biol Med (1990) 110(10):411–3. 1980623

[56] Yamabe T Takakura K Sugie K Kitaoka Y Takeda S Okubo Y. Induction of the 2B9 Antigen/Dipeptidyl Peptidase IV/CD26 on Human Natural Killer Cells by IL-2, IL-12 or IL-15. Immunology (1997) 91(1):151–8. doi: 10.1046/j.1365-2567.1997.00230.x PMC1364048 9203979

[57] Buhling F Reinhold D Lendeckel U Faust J Neubert K Ansorge S. CD26 is Involved in Regulation of Cytokine Production in Natural Killer Cells. Adv Exp Med Biol (1997) 421:141–7. doi: 10.1007/978-1-4757-9613-1_18 9330690

[58] Madueno JA Muñoz E Blazquez V Gonzalez R Aparicio P Peña J. The CD26 Antigen is Coupled to Protein Tyrosine Phosphorylation and Implicated in CD16-Mediated Lysis in Natural Killer Cells. Scand J Immunol (1993) 37(4):425–9. doi: 10.1111/j.1365-3083.1993.tb03313.x 8097057

[59] Schutz F Hackstein H. Identification of Novel Dendritic Cell Subset Markers in Human Blood. Biochem Biophys Res Commun (2014) 443(2):453–7. doi: 10.1016/j.bbrc.2013.11.112 24321550

[60] Nakano H Moran TP Nakano K Gerrish KE Bortner CD Cook DN. Complement Receptor C5aR1/CD88 and Dipeptidyl Peptidase-4/CD26 Define Distinct Hematopoietic Lineages of Dendritic Cells. J Immunol (2015) 194(8):3808–19. doi: 10.4049/jimmunol.1402195 PMC4390500 25769922

[61] Talker SC Baumann A Barut GT Keller I Bruggmann R Summerfield A. Precise Delineation and Transcriptional Characterization of Bovine Blood Dendritic-Cell and Monocyte Subsets. Front Immunol (2018) 9:2505. doi: 10.3389/fimmu.2018.02505 30425716 PMC6218925

[62] Ikedo T Minami M Kataoka H Hayashi K Nagata M Fujikawa R. Dipeptidyl Peptidase-4 Inhibitor Anagliptin Prevents Intracranial Aneurysm Growth by Suppressing Macrophage Infiltration and Activation. J Am Heart Assoc (2017) 6(6):e004777. doi: 10.1161/JAHA.116.004777 28630262 PMC5669147

[63] Ghorpade DS Ozcan L Zheng Z Nicoloro SM Shen Y Chen E. Hepatocyte-Secreted DPP4 in Obesity Promotes Adipose Inflammation and Insulin Resistance. Nature (2018) 555(7698):673–7. doi: 10.1038/nature26138 PMC6021131 29562231

[64] Zaruba MM Franz WM. Role of the SDF-1-CXCR4 Axis in Stem Cell-Based Therapies for Ischemic Cardiomyopathy. Expert Opin Biol Ther (2010) 10(3):321–35. doi: 10.1517/14712590903460286 20132055

[65] Aiuti A Webb IJ Bleul C Springer T Gutierrez-Ramos JC. The Chemokine SDF-1 is a Chemoattractant for Human CD34+ Hematopoietic Progenitor Cells and Provides a New Mechanism to Explain the Mobilization of CD34+ Progenitors to Peripheral Blood. J Exp Med (1997) 185(1):111–20. doi: 10.1084/jem.185.1.111 PMC2196104 8996247

[66] Kondo K Shintani S Shibata R Murakami H Murakami R Imaizumi M. Implantation of Adipose-Derived Regenerative Cells Enhances Ischemia-Induced Angiogenesis. Arterioscler Thromb Vasc Biol (2009) 29(1):61–6. doi: 10.1161/ATVBAHA.108.166496 18974384

[67] Janssens R Mortier A Boff D Ruytinx P Gouwy M Vantilt B. Truncation of CXCL12 by CD26 Reduces its CXC Chemokine Receptor 4- and Atypical Chemokine Receptor 3-Dependent Activity on Endothelial Cells and Lymphocytes. Biochem Pharmacol (2017) 132:92–101. doi: 10.1016/j.bcp.2017.03.009 28322746

[68] Herrera C Morimoto C Blanco J Mallol J Arenzana F Lluis C. Comodulation of CXCR4 and CD26 in Human Lymphocytes. J Biol Chem (2001) 276(22):19532–9. doi: 10.1074/jbc.M004586200 11278278

[69] Proost P Schutyser E Menten P Struyf S Wuyts A Opdenakker G. Amino-Terminal Truncation of CXCR3 Agonists Impairs Receptor Signaling and Lymphocyte Chemotaxis, While Preserving Antiangiogenic Properties. Blood (2001) 98(13):3554–61. doi: 10.1182/blood.V98.13.3554 11739156

[70] Barreira da Silva R Yatim N Fiette L Ingersoll MA Albert ML. Dipeptidylpeptidase 4 Inhibition Enhances Lymphocyte Trafficking, Improving Both Naturally Occurring Tumor Immunity and Immunotherapy. Nat Immunol (2015) 16(8):850–8. doi: 10.1038/ni.3201 26075911

[71] Hollande C Boussier J Ziai J Nozawa T Bondet V Phung W. Inhibition of the Dipeptidyl Peptidase DPP4 (CD26) Reveals IL-33-Dependent Eosinophil-Mediated Control of Tumor Growth. Nat Immunol (2019) 20(3):257–64. doi: 10.1038/s41590-019-0321-5 30778250

[72] Qin CJ Zhao LH Zhou X Zhang HL Wen W Tang L. Inhibition of Dipeptidyl Peptidase IV Prevents High Fat Diet-Induced Liver Cancer Angiogenesis by Downregulating Chemokine Ligand 2. Cancer Lett (2018) 420:26–37. doi: 10.1016/j.canlet.2018.01.064 29409972

[73] Proost P Menten P Struyf S Schutyser E De Meester I Van Damme J. Cleavage by CD26/dipeptidyl Peptidase IV Converts the Chemokine LD78beta Into a Most Efficient Monocyte Attractant and CCR1 Agonist. Blood (2000) 96(5):1674–80. doi: 10.1182/blood.V96.5.1674 10961862

[74] Struyf S Menten P Lenaerts JP Put W D'Haese A De Clercq E. Diverging Binding Capacities of Natural LD78beta Isoforms of Macrophage Inflammatory Protein-1alpha to the CC Chemokine Receptors 1, 3 and 5 Affect Their Anti-HIV-1 Activity and Chemotactic Potencies for Neutrophils and Eosinophils. Eur J Immunol (2001) 31(7):2170–8. doi: 10.1002/1521-4141(200107)31:7<2170::AID-IMMU2170>3.0.CO;2-D 11449371

[75] Oravecz T Pall M Roderiquez G Gorrell MD Ditto M Nguyen NY. Regulation of the Receptor Specificity and Function of the Chemokine RANTES (Regulated on Activation, Normal T Cell Expressed and Secreted) by Dipeptidyl Peptidase IV (CD26)-Mediated Cleavage. J Exp Med (1997) 186(11):1865–72. doi: 10.1084/jem.186.11.1865 PMC2199148 9382885

[76] Struyf S Proost P Schols D De Clercq E Opdenakker G Lenaerts JP. CD26/dipeptidyl-Peptidase IV Down-Regulates the Eosinophil Chemotactic Potency, But Not the Anti-HIV Activity of Human Eotaxin by Affecting its Interaction With CC Chemokine Receptor 3. J Immunol (1999) 162(8):4903–9. 10202035

[77] Proost P Struyf S Schols D Opdenakker G Sozzani S Allavena P. Truncation of Macrophage-Derived Chemokine by CD26/dipeptidyl-Peptidase IV Beyond its Predicted Cleavage Site Affects Chemotactic Activity and CC Chemokine Receptor 4 Interaction. J Biol Chem (1999) 274(7):3988–93. doi: 10.1074/jbc.274.7.3988 9933589

[78] Struyf S Proost P Sozzani S Mantovani A Wuyts A De Clercq E. Enhanced Anti-HIV-1 Activity and Altered Chemotactic Potency of NH2-Terminally Processed Macrophage-Derived Chemokine (MDC) Imply an Additional MDC Receptor. J Immunol (1998) 161(6):2672–5. 9743322

[79] Broxmeyer HE Hoggatt J O'Leary HA Mantel C Chitteti BR Cooper S. Dipeptidylpeptidase 4 Negatively Regulates Colony-Stimulating Factor Activity and Stress Hematopoiesis. Nat Med (2012) 18(12):1786–96. doi: 10.1038/nm.2991 PMC3742313 23160239

[80] Ou X O’Leary HA Broxmeyer HE. Implications of DPP4 Modification of Proteins That Regulate Stem/Progenitor and More Mature Cell Types. Blood (2013) 122(2):161–9. doi: 10.1182/blood-2013-02-487470 PMC3709652 23637126

[81] Wesley UV McGroarty M Homoyouni A. Dipeptidyl Peptidase Inhibits Malignant Phenotype of Prostate Cancer Cells by Blocking Basic Fibroblast Growth Factor Signaling Pathway. Cancer Res (2005) 65(4):1325–34. doi: 10.1158/0008-5472.CAN-04-1852 15735018

[82] Ahrén B Hughes TE. Inhibition of Dipeptidyl Peptidase-4 Augments Insulin Secretion in Response to Exogenously Administered Glucagon-Like Peptide-1, Glucose-Dependent Insulinotropic Polypeptide, Pituitary Adenylate Cyclase-Activating Polypeptide, and Gastrin-Releasing Peptide in Mice. Endocrinology (2005) 146(4):2055–9. doi: 10.1210/en.2004-1174 15604213

[83] Frerker N Wagner L Wolf R Heiser U Hoffmann T Rahfeld JU. Neuropeptide Y (NPY) Cleaving Enzymes: Structural and Functional Homologues of Dipeptidyl Peptidase 4. Peptides (2007) 28(2):257–68. doi: 10.1016/j.peptides.2006.09.027 17223229

[84] Unniappan S McIntosh CH Demuth HU Heiser U Wolf R Kieffer TJ. Effects of Dipeptidyl Peptidase IV on the Satiety Actions of Peptide YY. Diabetologia (2006) 49(8):1915–23. doi: 10.1007/s00125-006-0310-8 16802131

[85] Manuguerra-Gagne R Boulos PR Ammar A Leblond FA Krosl G Pichette V. Transplantation of Mesenchymal Stem Cells Promotes Tissue Regeneration in a Glaucoma Model Through Laser-Induced Paracrine Factor Secretion and Progenitor Cell Recruitment. Stem Cells (2013) 31(6):1136–48. doi: 10.1002/stem.1364 23495088

[86] Kalluri R Zeisberg M. Fibroblasts in Cancer. Nat Rev Cancer (2006) 6(5):392–401. doi: 10.1038/nrc1877 16572188

[87] Carmona-Rivera C Carlucci PM Moore E Lingampalli N Uchtenhagen H James E. Synovial Fibroblast-Neutrophil Interactions Promote Pathogenic Adaptive Immunity in Rheumatoid Arthritis. Sci Immunol (2017) 2(10):eaag3358. doi: 10.1126/sciimmunol.aag3358 28649674 PMC5479641

[88] Tran CN Davis MJ Tesmer LA Endres JL Motyl CD Smuda C. Presentation of Arthritogenic Peptide to Antigen-Specific T Cells by Fibroblast-Like Synoviocytes. Arthritis Rheum (2007) 56(5):1497–506. doi: 10.1002/art.22573 17469112

[89] Henderson NC Rieder F Wynn TA. Fibrosis: From Mechanisms to Medicines. Nature (2020) 587(7835):555–66. doi: 10.1038/s41586-020-2938-9 PMC8034822 33239795

[90] Tabib T Morse C Wang T Chen W Lafyatis R. SFRP2/DPP4 and FMO1/LSP1 Define Major Fibroblast Populations in Human Skin. J Invest Dermatol (2018) 138(4):802–10. doi: 10.1016/j.jid.2017.09.045 PMC7444611 29080679

[91] Soare A Györfi HA Matei AE Dees C Rauber S Wohlfahrt T. Dipeptidylpeptidase 4 as a Marker of Activated Fibroblasts and a Potential Target for the Treatment of Fibrosis in Systemic Sclerosis. Arthritis Rheumatol (2020) 72(1):137–49. doi: 10.1002/art.41058 31350829

[92] Lee SY Wu ST Liang YJ Su MJ Huang CW Jao YH. Soluble Dipeptidyl Peptidase-4 Induces Fibroblast Activation Through Proteinase-Activated Receptor-2. Front Pharmacol (2020) 11:552818. doi: 10.3389/fphar.2020.552818 33117158 PMC7561399

[93] Li YC Sung PH Yang YH Chiang JY Yip HK Yang CC. Dipeptidyl Peptidase 4 Promotes Peritoneal Fibrosis and its Inhibitions Prevent Failure of Peritoneal Dialysis. Commun Biol (2021) 4(1):144. doi: 10.1038/s42003-021-01652-x 33514826 PMC7846859

[94] Löster K Zeilinger K Schuppan D Reutter W. The Cysteine-Rich Region of Dipeptidyl Peptidase IV (CD 26) is the Collagen-Binding Site. Biochem Biophys Res Commun (1995) 217(1):341–8. doi: 10.1006/bbrc.1995.2782 8526932

[95] Cheng HC Abdel-Ghany M Pauli BU. A Novel Consensus Motif in Fibronectin Mediates Dipeptidyl Peptidase IV Adhesion and Metastasis. J Biol Chem (2003) 278(27):24600–7. doi: 10.1074/jbc.M303424200 12716896

[96] Gonzalez-Gronow M Kaczowka S Gawdi G Pizzo SV. Dipeptidyl Peptidase IV (DPP IV/CD26) is a Cell-Surface Plasminogen Receptor. Front Biosci (2008) 13:1610–8. doi: 10.2741/2785 17981653

[97] Gonzalez-Gronow M Kaczowka S Gawdi G Pizzo SV. Interaction of Plasminogen With Dipeptidyl Peptidase IV Initiates a Signal Transduction Mechanism Which Regulates Expression of Matrix Metalloproteinase-9 by Prostate Cancer Cells. Biochem J (2001) 355(Pt 2):397–407. doi: 10.1042/bj3550397 11284727 PMC1221751

[98] Shao S Xu Q Yu X Pan R Chen Y. Dipeptidyl Peptidase 4 Inhibitors and Their Potential Immune Modulatory Functions. Pharmacol Ther (2020) 209:107503. doi: 10.1016/j.pharmthera.2020.107503 32061923 PMC7102585

[99] Panayi GS Lanchbury JS Kingsley GH. The Importance of the T Cell in Initiating and Maintaining the Chronic Synovitis of Rheumatoid Arthritis. Arthritis Rheum (1992) 35(7):729–35. doi: 10.1002/art.1780350702 1622409

[100] McInnes IB Schett G. The Pathogenesis of Rheumatoid Arthritis. N Engl J Med (2011) 365(23):2205–19. doi: 10.1056/NEJMra1004965 22150039

[101] Sinnathurai P Lau W Vieira de Ribeiro AJ Bachovchin WW Englert H Howe G. Circulating Fibroblast Activation Protein and Dipeptidyl Peptidase 4 in Rheumatoid Arthritis and Systemic Sclerosis. Int J Rheum Dis (2018) 21(11):1915–23. doi: 10.1111/1756-185X.13031 PMC5476518 27990763

[102] Cordero OJ Salgado FJ Mera-Varela A Nogueira M. Serum Interleukin-12, Interleukin-15, Soluble CD26, and Adenosine Deaminase in Patients With Rheumatoid Arthritis. Rheumatol Int (2001) 21(2):69–74. doi: 10.1007/s002960100134 11732862

[103] Grujic M Matic IZ Crnogorac MD Velickovic AD Kolundzija B Cordero OJ. Activity and Expression of Dipeptidyl Peptidase IV on Peripheral Blood Mononuclear Cells in Patients With Early Steroid and Disease Modifying Antirheumatic Drugs Naive Rheumatoid Arthritis. Clin Chem Lab Med (2017) 55(1):73–81. doi: 10.1515/cclm-2015-1279 27341562

[104] Muscat C Bertotto A Agea E Bistoni O Ercolani R Tognellini R. Expression and Functional Role of 1F7 (CD26) Antigen on Peripheral Blood and Synovial Fluid T Cells in Rheumatoid Arthritis Patients. Clin Exp Immunol (1994) 98(2):252–6. doi: 10.1111/j.1365-2249.1994.tb06134.x PMC1534402 7955530

[105] Sromova L Mareckova H Sedova L Balaziova E Sedo A. Dipeptidyl Peptidase-IV in Synovial Fluid and in Synovial Fluid Mononuclear Cells of Patients With Rheumatoid Arthritis. Clin Chim Acta (2010) 411(15-16):1046–50. doi: 10.1016/j.cca.2010.03.034 20361950

[106] Buljevic S Detel D Pucar LB Mihelic R Madarevic T Sestan B. Levels of Dipeptidyl Peptidase IV/CD26 Substrates Neuropeptide Y and Vasoactive Intestinal Peptide in Rheumatoid Arthritis Patients. Rheumatol Int (2013) 33(11):2867–74. doi: 10.1007/s00296-013-2823-z 23864142

[107] Kamori M Hagihara M Nagatsu T Iwata H Miura T. Activities of Dipeptidyl Peptidase II, Dipeptidyl Peptidase IV, Prolyl Endopeptidase, and Collagenase-Like Peptidase in Synovial Membrane From Patients With Rheumatoid Arthritis and Osteoarthritis. Biochem Med Metab Biol (1991) 45(2):154–60. doi: 10.1016/0885-4505(91)90016-E 1679339

[108] Cordero OJ Varela-Calviño R López-González T Grujic M Juranic Z Mouriño C. Anti-CD26 Autoantibodies are Involved in Rheumatoid Arthritis and Show Potential Clinical Interest. Clin Biochem (2017) 50(16-17):903–10. doi: 10.1016/j.clinbiochem.2017.06.001 28599787

[109] Busso N Wagtmann N Herling C Chobaz-Péclat V Bischof-Delaloye A So A. Circulating CD26 is Negatively Associated With Inflammation in Human and Experimental Arthritis. Am J Pathol (2005) 166(2):433–42. doi: 10.1016/S0002-9440(10)62266-3 PMC1602320 15681827

[110] Kanbe K Chiba J Inoue Y Taguchi M Yabuki A. SDF-1 and CXCR4 in Synovium are Associated With Disease Activity and Bone and Joint Destruction in Patients With Rheumatoid Arthritis Treated With Golimumab. Mod Rheumatol (2016) 26(1):46–50. doi: 10.3109/14397595.2015.1054088 25995033

[111] Han CK Lee WF Hsu CJ Huang YL Lin CY Tsai CH. DPP4 Reduces Proinflammatory Cytokine Production in Human Rheumatoid Arthritis Synovial Fibroblasts. J Cell Physiol (2021) 236(12):8060–9. doi: 10.1002/jcp.30494 34192347

[112] Kanbe K Chiba J Inoue Y Taguchi M Yabuki A. Identification of Osteoclast-Osteoblast Coupling Factors in Humans Reveals Links Between Bone and Energy Metabolism. Nat Commun (2020) 11(1):87. doi: 10.1038/s41467-019-14003-6 31911667 PMC6946812

[113] Ospelt C Mertens JC Jüngel A Brentano F Maciejewska-Rodriguez H Huber LC. Inhibition of Fibroblast Activation Protein and Dipeptidylpeptidase 4 Increases Cartilage Invasion by Rheumatoid Arthritis Synovial Fibroblasts. Arthritis Rheum (2010) 62(5):1224–35. doi: 10.1002/art.27395 20155839

[114] Glorie L Behets GJ Baerts L De Meester I D'Haese PC Verhulst A. DPP IV Inhibitor Treatment Attenuates Bone Loss and Improves Mechanical Bone Strength in Male Diabetic Rats. Am J Physiol Endocrinol Metab (2014) 307(5):E447–55. doi: 10.1152/ajpendo.00217.2014 25053403

[115] Yokota K Igaki N. Sitagliptin (DPP-4 Inhibitor)-Induced Rheumatoid Arthritis in Type 2 Diabetes Mellitus: A Case Report. Intern Med (2012) 51(15):2041–4. doi: 10.2169/internalmedicine.51.7592 22864134

[116] Padron S Rogers E Demory Beckler M Kesselman M. DPP-4 Inhibitor (Sitagliptin)-Induced Seronegative Rheumatoid Arthritis. BMJ Case Rep (2019) 12(8):e228981. doi: 10.1136/bcr-2018-228981 PMC6720914 31444259

[117] Mascolo A Rafaniello C Sportiello L Sessa M Cimmaruta D Rossi F. Dipeptidyl Peptidase (DPP)-4 Inhibitor-Induced Arthritis/Arthralgia: A Review of Clinical Cases. Drug Saf (2016) 39(5):401–7. doi: 10.1007/s40264-016-0399-8 26873369

[118] Kathe N Shah A Said Q Painter JT. DPP-4 Inhibitor-Induced Rheumatoid Arthritis Among Diabetics: A Nested Case-Control Study. Diabetes Ther (2018) 9(1):141–51. doi: 10.1007/s13300-017-0353-5 PMC5801239 29236221

[119] Douros A Abrahami D Yin H Yu OHY Renoux C Hudson M. Use of Dipeptidyl Peptidase-4 Inhibitors and New-Onset Rheumatoid Arthritis in Patients With Type 2 Diabetes. Epidemiology (2018) 29(6):904–12. doi: 10.1097/EDE.0000000000000891 30028343

[120] Charoenngam N Rittiphairoj T Ponvilawan B Ungprasert P. Use of Dipeptidyl Peptidase-4 Inhibitors is Associated With a Lower Risk of Rheumatoid Arthritis in Patients With Type 2 Diabetes Mellitus: A Systematic Review and Meta-Analysis of Cohort Studies. Diabetes Metab Syndr (2021) 15(1):249–55. doi: 10.1016/j.dsx.2020.12.042 33465685

[121] Wang M Li M Xie Y. Systematic Review and Meta-Analysis: Dipeptidyl Peptidase-4 Inhibitors and Rheumatoid Arthritis Risk. Endocr J (2021) 68(6):729–38. doi: 10.1507/endocrj.EJ20-0647 33642418

[122] Dema B Charles N. Advances in Mechanisms of Systemic Lupus Erythematosus. Discov Med (2014) 17(95):247–55. 24882716

[123] StancÍková M Lojda Z Lukác J Ruzicková M. Dipeptidyl Peptidase IV in Patients With Systemic Lupus Erythematosus. Clin Exp Rheumatol (1992) 10(4):381–5. 1356680

[124] Kobayashi H Hosono O Mimori T Kawasaki H Dang NH Tanaka H. Reduction of Serum Soluble CD26/dipeptidyl Peptidase IV Enzyme Activity and its Correlation With Disease Activity in Systemic Lupus Erythematosus. J Rheumatol (2002) 29(9):1858–66. 12233879

[125] Wong PT Wong CK Tam LS Li EK Chen DP Lam CW. Decreased Expression of T Lymphocyte Co-Stimulatory Molecule CD26 on Invariant Natural Killer T Cells in Systemic Lupus Erythematosus. Immunol Invest (2009) 38(5):350–64. doi: 10.1080/08820130902770003 19811413

[126] Seong JM Yee J Gwak HS. Dipeptidyl Peptidase-4 Inhibitors Lower the Risk of Autoimmune Disease in Patients With Type 2 Diabetes Mellitus: A Nationwide Population-Based Cohort Study. Br J Clin Pharmacol (2019) 85(8):1719–27. doi: 10.1111/bcp.13955 PMC6624390 30964554

[127] Chen YC Chen TH Sun CC Chen JY Chang SS Yeung L. Dipeptidyl Peptidase-4 Inhibitors and the Risks of Autoimmune Diseases in Type 2 Diabetes Mellitus Patients in Taiwan: A Nationwide Population-Based Cohort Study. Acta Diabetol (2020) 57(10):1181–92. doi: 10.1007/s00592-020-01533-5 PMC7173685 32318876

[128] Denton CP Khanna D. Systemic Sclerosis. Lancet (2017) 390(10103):1685–99. doi: 10.1016/S0140-6736(17)30933-9 28413064

[129] Fiocco U Rosada M Cozzi L Ortolani C De Silvestro G Ruffatti A. Early Phenotypic Activation of Circulating Helper Memory T Cells in Scleroderma: Correlation With Disease Activity. Ann Rheum Dis (1993) 52(4):272–7. doi: 10.1136/ard.52.4.272 PMC1005625 8484693

[130] Tamaki Z Kubo M Yazawa N Mimura Y Ashida R Tomita M. Serum Levels of Soluble CD26 in Patients With Scleroderma. J Dermatol Sci (2008) 52(1):67–9. doi: 10.1016/j.jdermsci.2008.05.004 18595667

[131] Hinz B. Myofibroblasts. Exp Eye Res (2016) 142:56–70. doi: 10.1016/j.exer.2015.07.009 26192991

[132] Kissin EY Merkel PA Lafyatis R. Myofibroblasts and Hyalinized Collagen as Markers of Skin Disease in Systemic Sclerosis. Arthritis Rheum (2006) 54(11):3655–60. doi: 10.1002/art.22186 17075814

[133] Ziemek J Man A Hinchcliff M Varga J Simms RW Lafyatis R. The Relationship Between Skin Symptoms and the Scleroderma Modification of the Health Assessment Questionnaire, the Modified Rodnan Skin Score, and Skin Pathology in Patients With Systemic Sclerosis. Rheumatol (Oxford) (2016) 55(5):911–7. doi: 10.1093/rheumatology/kew003 PMC5854039 26880832

[134] Xin Y Wang X Zhu M Qu M Bogari M Lin L. Expansion of CD26 Positive Fibroblast Population Promotes Keloid Progression. Exp Cell Res (2017) 356(1):104–13. doi: 10.1016/j.yexcr.2017.04.021 28454879

[135] Shiobara T Chibana K Watanabe T Arai R Horigane Y Nakamura Y. Dipeptidyl Peptidase-4 is Highly Expressed in Bronchial Epithelial Cells of Untreated Asthma and it Increases Cell Proliferation Along With Fibronectin Production in Airway Constitutive Cells. Respir Res (2016) 17:28. doi: 10.1186/s12931-016-0342-7 26975422 PMC4791890

[136] Tabib T Huang M Morse N Papazoglou A Behera R Jia M. Myofibroblast Transcriptome Indicates SFRP2(hi) Fibroblast Progenitors in Systemic Sclerosis Skin. Nat Commun (2021) 12(1):4384. doi: 10.1038/s41467-021-24607-6 34282151 PMC8289865

[137] Liu Y Qi Y. Vildagliptin, a CD26/DPP4 Inhibitor, Ameliorates Bleomycin-Induced Pulmonary Fibrosis *via* Regulating the Extracellular Matrix. Int Immunopharmacol (2020) 87:106774. doi: 10.1016/j.intimp.2020.106774 32731178

[138] Hildebrandt M Rose M Rüter J Salama A Mönnikes H Klapp BF. Dipeptidyl Peptidase IV (DP IV, CD26) in Patients With Inflammatory Bowel Disease. Scand J Gastroenterol (2001) 36(10):1067–72. doi: 10.1080/003655201750422675 11589380

[139] Pinto-Lopes P Afonso J Pinto-Lopes R Rocha C Lago P Gonçalves R. Serum Dipeptidyl Peptidase 4: A Predictor of Disease Activity and Prognosis in Inflammatory Bowel Disease. Inflamm Bowel Dis (2020) 26(11):1707–19. doi: 10.1093/ibd/izz319 31912883

[140] Magro DO Kotze PG Martinez CAR Camargo MG Guadagnini D Calixto AR. Changes in Serum Levels of Lipopolysaccharides and CD26 in Patients With Crohn’s Disease. Intest Res (2017) 15(3):352–7. doi: 10.5217/ir.2017.15.3.352 PMC5478760 28670232

[141] Moran GW O'Neill C Padfield P McLaughlin JT. Dipeptidyl Peptidase-4 Expression is Reduced in Crohn’s Disease. Regul Pept (2012) 177(1-3):40–5. doi: 10.1016/j.regpep.2012.04.006 22561447

[142] Pinto-Lopes P Melo F Afonso J Pinto-Lopes R Rocha C Melo D. Fecal Dipeptidyl Peptidase-4: An Emergent Biomarker in Inflammatory Bowel Disease. Clin Transl Gastroenterol (2021) 12(3):e00320. doi: 10.14309/ctg.0000000000000320 33704099 PMC7954374

[143] Melo FJ Pinto-Lopes P Estevinho MM Magro F. The Role of Dipeptidyl Peptidase 4 as a Therapeutic Target and Serum Biomarker in Inflammatory Bowel Disease: A Systematic Review. Inflammation Bowel Dis (2021) 27(7):1153–65. doi: 10.1093/ibd/izaa324 33295607

[144] Baticic L. Neuroimmunomodulative Properties of Dipeptidyl Peptidase IV/CD26 in a TNBS-Induced Model of Colitis in Mice. J Cell Biochem (2011) 112(11):3322–33. doi: 10.1002/jcb.23261 21751235

[145] Detel D Detel D Kucic N Buljevic S Pugel EP Varljen J. Influence of CD26/dipeptidyl Peptidase IV Deficiency on Immunophenotypic Changes During Colitis Development and Resolution. J Physiol Biochem (2016) 72(3):405–19. doi: 10.1007/s13105-016-0491-7 27125676

[146] Duan L Rao X Braunstein Z Toomey AC Zhong J. Role of Incretin Axis in Inflammatory Bowel Disease. Front Immunol (2017) 8:1734. doi: 10.3389/fimmu.2017.01734 29270177 PMC5723660

[147] Ning MM Yang WJ Guan WB Gu YP Feng Y Leng Y. Dipeptidyl Peptidase 4 Inhibitor Sitagliptin Protected Against Dextran Sulfate Sodium-Induced Experimental Colitis by Potentiating the Action of GLP-2. Acta Pharmacol Sin (2020) 41(11):1446–56. doi: 10.1038/s41401-020-0413-7 PMC7656800 32398684

[148] Ban H Bamba S Imaeda H Inatomi O Kobori A Sasaki M. The DPP-IV Inhibitor ER-319711 has a Proliferative Effect on the Colonic Epithelium and a Minimal Effect in the Amelioration of Colitis. Oncol Rep (2011) 25(6):1699–703. doi: 10.3892/or.2011.1223 21431278

[149] Mimura S Ando T Ishiguro K Maeda O Watanabe O Ujihara M. Dipeptidyl Peptidase-4 Inhibitor Anagliptin Facilitates Restoration of Dextran Sulfate Sodium-Induced Colitis. Scand J Gastroenterol (2013) 48(10):1152–9. doi: 10.3109/00365521.2013.832366 24047394

[150] Kridin K Amber K Khamaisi M Comaneshter D Batat E Cohen AD. Is There an Association Between Dipeptidyl Peptidase-4 Inhibitors and Autoimmune Disease? A Population-Based Study. Immunol Res (2018) 66(3):425–30. doi: 10.1007/s12026-018-9005-8 29855994

[151] Abrahami D Douros A Yin H Yu OHY Renoux C Bitton A. Dipeptidyl Peptidase-4 Inhibitors and Incidence of Inflammatory Bowel Disease Among Patients With Type 2 Diabetes: Population Based Cohort Study. BMJ (2018) 360:k872. doi: 10.1136/bmj.k872 29563098 PMC5861502

[152] Wang T Yang JY Buse JB Pate V Tang H Barnes EL. Dipeptidyl Peptidase 4 Inhibitors and Risk of Inflammatory Bowel Disease: Real-World Evidence in U.S. Adults. Diabetes Care (2019) 42(11):2065–74. doi: 10.2337/dc19-0162 PMC6804610 31471377

[153] Radel JA Pender DN Shah SA. Dipeptidyl Peptidase-4 Inhibitors and Inflammatory Bowel Disease Risk: A Meta-Analysis. Ann Pharmacother (2019) 53(7):697–704. doi: 10.1177/1060028019827852 30700100

[154] Roep BO Tree TIM. Immune Modulation in Humans: Implications for Type 1 Diabetes Mellitus. Nat Rev Endocrinol (2014) 10(4):229–42. doi: 10.1038/nrendo.2014.2 24468651

[155] Iwabuchi A Kamoda T Saito M Nozue H Izumi I Hirano T. Serum Dipeptidyl Peptidase 4 Activity in Children With Type 1 Diabetes Mellitus. J Pediatr Endocrinol Metab (2013) 26(11-12):1093–7. doi: 10.1515/jpem-2013-0122 23817599

[156] Varga T Somogyi A Barna G Wichmann B Nagy G Rácz K. Higher Serum DPP-4 Enzyme Activity and Decreased Lymphocyte CD26 Expression in Type 1 Diabetes. Pathol Oncol Res (2011) 17(4):925–30. doi: 10.1007/s12253-011-9404-9 21785903

[157] Osawa S Kawamori D Katakami N Takahara M Sakamoto F Katsura T. Significant Elevation of Serum Dipeptidyl Peptidase-4 Activity in Young-Adult Type 1 Diabetes. Diabetes Res Clin Pract (2016) 113:135–42. doi: 10.1016/j.diabres.2015.12.022 26827118

[158] Ellis SL Snell-Bergeon JK Rodionova AS Hazenfield RM Garg SK. Effect of Sitagliptin on Glucose Control in Adult Patients With Type 1 Diabetes: A Pilot, Double-Blind, Randomized, Crossover Trial. Diabetes Med (2011) 28(10):1176–81. doi: 10.1111/j.1464-5491.2011.03331.x 21923696

[159] Farngren J Persson M Schweizer A Foley JE Ahrén B. Vildagliptin Reduces Glucagon During Hyperglycemia and Sustains Glucagon Counterregulation During Hypoglycemia in Type 1 Diabetes. J Clin Endocrinol Metab (2012) 97(10):3799–806. doi: 10.1210/jc.2012-2332 22855332

[160] Garg SK Moser EG Bode BW Klaff LJ Hiatt WR Beatson C. Effect of Sitagliptin on Post-Prandial Glucagon and GLP-1 Levels in Patients With Type 1 Diabetes: Investigator-Initiated, Double-Blind, Randomized, Placebo-Controlled Trial. Endocr Pract (2013) 19(1):19–28. doi: 10.4158/EP12100.OR 23186950

[161] Hari Kumar KV Shaikh A Prusty P. Addition of Exenatide or Sitagliptin to Insulin in New Onset Type 1 Diabetes: A Randomized, Open Label Study. Diabetes Res Clin Pract (2013) 100(2):e55–8. doi: 10.1016/j.diabres.2013.01.020 23490599

[162] Cechin SR Pérez-Álvarez I Fenjves E Molano RD Pileggi A Berggren PO. Anti-Inflammatory Properties of Exenatide in Human Pancreatic Islets. Cell Transplant (2012) 21(4):633–48. doi: 10.3727/096368911X576027 21669040

[163] Kim SJ Nian C Doudet DJ McIntosh CH. Dipeptidyl Peptidase IV Inhibition With MK0431 Improves Islet Graft Survival in Diabetic NOD Mice Partially *via* T-Cell Modulation. Diabetes (2009) 58(3):641–51. doi: 10.2337/db08-1101 PMC2646063 19073764

[164] Davanso MR Caliari-Oliveira C Couri CEB Covas DT de Oliveira Leal AM Voltarelli JC. DPP-4 Inhibition Leads to Decreased Pancreatic Inflammatory Profile and Increased Frequency of Regulatory T Cells in Experimental Type 1 Diabetes. Inflammation (2019) 42(2):449–62. doi: 10.1007/s10753-018-00954-3 30707388

[165] Tejera-Alhambra M Casrouge A de Andrés C Ramos-Medina R Alonso B Vega J. Low DPP4 Expression and Activity in Multiple Sclerosis. Clin Immunol (2014) 150(2):170–83. doi: 10.1016/j.clim.2013.11.011 24412911

[166] Garreto L Charneau S Mandacaru SC Nóbrega OT Motta FN de Araújo CN. Mapping Salivary Proteases in Sjogren’s Syndrome Patients Reveals Overexpression of Dipeptidyl Peptidase-4/Cd26. Front Immunol (2021) 12:686480. doi: 10.3389/fimmu.2021.686480 34220840 PMC8247581

[167] Solau-Gervais E Zerimech F Lemaire R Fontaine C Huet G Flipo RM. Cysteine and Serine Proteases of Synovial Tissue in Rheumatoid Arthritis and Osteoarthritis. Scand J Rheumatol (2007) 36(5):373–7. doi: 10.1080/03009740701340172 17963167

[168] Hu N Gong X Yin S Li Q Chen H Li Y. Saxagliptin Suppresses Degradation of Type II Collagen and Aggrecan in Primary Human Chondrocytes: A Therapeutic Implication in Osteoarthritis. Artif Cells Nanomed Biotechnol (2019) 47(1):3239–45. doi: 10.1080/21691401.2019.1647223 31364869

[169] Gao F Wang Y Wu M. Teneligliptin Inhibits IL-1beta-Induced Degradation of Extracellular Matrix in Human Chondrocytes. J Cell Biochem (2020) 121(11):4450–7. doi: 10.1002/jcb.29662 32162384

[170] van Lingen RG van de Kerkhof PC Seyger MM de Jong EM van Rens DW Poll MK. CD26/dipeptidyl-Peptidase IV in Psoriatic Skin: Upregulation and Topographical Changes. Br J Dermatol (2008) 158(6):1264–72. doi: 10.1111/j.1365-2133.2008.08515.x 18384439

[171] Nishioka T Shinohara M Tanimoto N Kumagai C Hashimoto K. Sitagliptin, a Dipeptidyl Peptidase-IV Inhibitor, Improves Psoriasis. Dermatology (2012) 224(1):20–1. doi: 10.1159/000333358 22056790

[172] Lynch M Malara A Timoney I Vencken S Ahern T Awdeh F. Sitagliptin and Narrow-Band Ultraviolet-B for Moderate Psoriasis (DINUP): A Randomised Controlled Clinical Trial. Dermatology (2021) p:1–8. doi: 10.1159/000514494 33866313

[173] Guo JY Chen HH Yang YC Wu PY Chang MP Chen CC. The Association of Dipeptidyl Peptidase IV Inhibitors and Other Risk Factors With Bullous Pemphigoid in Patients With Type 2 Diabetes Mellitus: A Retrospective Cohort Study. J Diabetes Complicat (2020) 34(3):107515. doi: 10.1016/j.jdiacomp.2019.107515 31932172

[174] Tasanen K Varpuluoma O Nishie W. Dipeptidyl Peptidase-4 Inhibitor-Associated Bullous Pemphigoid. Front Immunol (2019) 10:1238. doi: 10.3389/fimmu.2019.01238 31275298 PMC6593303

[175] Aouidad I Fite C Marinho E Deschamps L Crickx B Descamps V. A Case Report of Bullous Pemphigoid Induced by Dipeptidyl Peptidase-4 Inhibitors. JAMA Dermatol (2013) 149(2):243–5. doi: 10.1001/jamadermatol.2013.1073 23426497

[176] Lee SG Lee HJ Yoon MS Kim DH. Association of Dipeptidyl Peptidase 4 Inhibitor Use With Risk of Bullous Pemphigoid in Patients With Diabetes. JAMA Dermatol (2019) 155(2):172–7. doi: 10.1001/jamadermatol.2018.4556 PMC6439542 30624566

[177] Kridin K Bergman R. Association of Bullous Pemphigoid With Dipeptidyl-Peptidase 4 Inhibitors in Patients With Diabetes: Estimating the Risk of the New Agents and Characterizing the Patients. JAMA Dermatol (2018) 154(10):1152–8. doi: 10.1001/jamadermatol.2018.2352 PMC6233738 30090931

[178] Zhang P Chen Y Zhao H Du H. Protective Effects of Alogliptin Against TNF-Alpha-Induced Degradation of Extracellular Matrix in Human Chondrocytes. Int Immunopharmacol (2019) 68:179–84. doi: 10.1016/j.intimp.2018.11.007 30654307

[179] Zhu S Gu Y Wang W Bai J Ge G Zhang W. Sitagliptin Ameliorates Advanced Glycation End-Product (AGE)-Induced Degradation of Extracellular Matrix in Human Primary Chondrocytes. Am J Transl Res (2019) 11(5):2775–83. PMC6556674 31217853

[180] Nargis T Kumar K Ghosh AR Sharma A Rudra D Sen D. KLK5 Induces Shedding of DPP4 From Circulatory Th17 Cells in Type 2 Diabetes. Mol Metab (2017) 6(11):1529–39. doi: 10.1016/j.molmet.2017.09.004 PMC5681279 29107298

[181] Wang Z Grigo C Steinbeck J von Hörsten S Amann K. Soluble DPP4 Originates in Part From Bone Marrow Cells and Not From the Kidney. Peptides (2014) 57:109–17. doi: 10.1016/j.peptides.2014.05.006 24874705

